# Using Smart Displays to Implement an eHealth System for Older Adults With Multiple Chronic Conditions: Randomized Controlled Trial

**DOI:** 10.2196/75991

**Published:** 2025-11-18

**Authors:** Gina Landucci, David H Gustafson Sr, Marie-Louise Mares, Klaren Pe-Romashko, John J Curtin, Yaxin Hu, Adam Maus, Kasey Thompson, Sydney Saunders, Kaitlyn Brown, Judith Woodburn, Bilge Mutlu

**Affiliations:** 1 Center for Health Enhancement Systems Studies, University of Wisconsin–Madison Madison, WI United States; 2 Department of Communication Arts, University of Wisconsin–Madison Madison, WI United States; 3 Department of Psychology, University of Wisconsin–Madison Madison, WI United States; 4 Department of Computer Sciences, University of Wisconsin–Madison Madison, WI United States

**Keywords:** aged, chronic pain, eHealth, geriatrics, health expenditures, multiple chronic conditions, primary care, quality of life, smart displays, smart speakers

## Abstract

**Background:**

Smart displays and speakers offer voice interaction, which may be more accessible and appealing to older adults with chronic pain and other multimorbid conditions. Previous trials found stronger socioemotional benefits of ElderTree (vs control) among those with high primary care use and multiple chronic conditions.

**Objective:**

This study aims to test whether older adults with chronic pain and multiple other chronic conditions use and benefit more from ElderTree, an eHealth intervention targeting pain and quality of life, when delivered on a smart display.

**Methods:**

We recruited 269 participants from the University of Wisconsin-Madison health system and community organizations and randomly assigned 1:1:1 to (1) smart display with internet and ElderTree, plus usual care; (2) touchscreen laptop with internet and ElderTree, plus usual care; or (3) usual care alone. Participants were aged ≥60 years, had a chronic pain diagnosis or reported chronic pain, and at least 3 common chronic conditions. Primary outcomes were pain interference and psychosocial quality of life. Data sources were baseline, 4-month, and 8-month surveys and continuous ElderTree usage data.

**Results:**

No significant differences were found between the laptop versus smart display groups for pain interference (b=–0.11, 95% CI –1.07 to 0.85; *P*=.82) or psychosocial quality (b=–0.21, 95% CI –0.96 to 0.55; *P*=.56), nor between the combined laptop+smart display group versus control group for either outcome (pain interference: b=–0.41, 95% CI –1.23 to 0.41; *P*=.33; psychosocial quality of life: b=0.04, 95% CI –0.61 to 0.69; *P*=.90). Mediation was not tested because effects on primary outcomes were nonsignificant. Gender did not moderate the effect of laptop versus smart display groups in pain interference (b=–1.56, 95% CI –3.56 to 0.44; *P*=.13). Gender did moderate the effect of the combined laptop+smart display group versus control group (b=1.91, 95% CI 0.11 to 3.71; *P*=.04). Women showed a significant decrease in pain interference (b=–0.69, 95% CI –1.29 to –0.10; *P*=.02), whereas women in the control group showed no significant change (b=0.25, 95% CI –0.53 to 1.04; *P*=.53). Men in the combined group showed a nonsignificant decrease (b=–0.67, 95% CI –1.47 to 0.14; *P*=.10), whereas men in the control group showed a significant decrease (b=–1.61, 95% CI –2.88 to –0.35; *P*=.01). Participants assigned to the laptop versus smart display used ElderTree more frequently and had more favorable perceptions. Analyses of secondary and exploratory outcomes showed no significant differences between groups.

**Conclusions:**

We found no significant differences between the combined ElderTree group and the control group for changes over time in any primary, secondary, or exploratory outcomes. Moderation analyses indicated that only gender moderated study arm effects, and only for the laptop+smart display versus control group on changes over time in the two primary outcomes.

**Trial Registration:**

ClinicalTrials.gov NCT04798196; https://clinicaltrials.gov/ct2/show/NCT04798196

**International Registered Report Identifier (IRRID):**

RR2-10.2196/37522

## Introduction

### Background

This study reports the outcomes of a randomized controlled trial examining the uses and effects of an 8-month eHealth intervention for older adults, delivered through two platforms—laptop computer versus smart display—compared with a no-exposure control group. To our knowledge, this is one of the first large trials to compare these platforms for delivering a health intervention. As discussed below, studies of smart device interventions for health outcomes have typically not involved random assignment, comparisons with other platforms, or large sample sizes.

We focused on older adults experiencing chronic pain with multiple other chronic conditions. The intervention was designed to help them understand and manage their pain in the context of complex health challenges. As such, this was an intervention for individuals expected to desire relief but who might face challenges in achieving it. Chronic pain not only affects mobility [1,2], but is also associated with reductions in attention, working memory capacity [3], and executive function [4]. Easy, engaging methods of interacting with an intervention may be particularly important under these circumstances. Given this expectation, the core hypotheses were that the intervention would be more effective when delivered on a smart display than on a laptop, and that both platforms would outperform the control group on the primary outcomes of pain interference and quality of life.

### Voice Interactivity and Smart Devices

Like laptops, smart displays are compact, internet-connected computers. A key difference, as reviewed in our protocol paper [5], is the supposed ease of voice interactivity. Users of smart displays can ask questions and give commands by speaking rather than typing and can hear rather than read the device’s response. Laptops, on the other hand, are primarily designed for typing and reading. Moreover, chatbot functions, now rapidly evolving with progress in artificial intelligence (AI) and voice recognition, allow the audio interactions of smart displays to, to varying degrees, approximate human conversations. Smart speakers offer the same voice interactivity as smart displays, but the latter have the added advantage of a screen that supports touch navigation of visual menus and video viewing.

Various researchers have proposed that the voice interactivity of smart speakers and smart displays may be particularly accessible and appealing for older adults [6-11], and these claims have been echoed in mass media discussions of voice-interactive devices [12]. Proposed advantages of voice interactivity include compensating for mobility issues that make it difficult to walk across a room to a computer, hand tremors or arthritis that make typing or controlling a cursor challenging, and vision loss that makes reading text or deciphering small images difficult. An additional possibility is that voice interactivity may enhance feelings of companionship, particularly as natural language processing improves the quality of smart devices’ responses. Relatedly, voice interactivity may offer easier, more intuitive access to content than computers or mobile phones, which require knowledge of where to tap or click, how to scroll, and how to bookmark content. Because smart speakers and smart displays are used with short vocal prompts, they have been suggested as better suited than computers or even smartphones to support older adults’ health and well-being [6,7,13,14].

Researchers have also noted potential difficulties with voice interactivity for older adults [15-17]. Age-related physical changes may reduce the volume, pace, and clarity of older adults’ speech, which in turn reduce the accuracy of voice-activated devices in interpreting their commands or questions. Hearing loss may impair the ability to understand the device’s responses. Smart systems that require memorizing specific commands or “skills” may not be easy to master. Older adults, like others, may also have concerns about privacy and surveillance, including the possibility of accidentally activating the speech-controlled device.

Current evidence on the benefits and challenges of voice-activated smart systems for older adults is somewhat limited. A 2024 scoping review of studies published between 2010 and 2020 on community-dwelling older adults and “personal voice assistants” identified 22 studies with a total of 284 participants [18]. All 22 studies were exploratory examinations of older adults’ use and acceptance of these technologies and none examined effects on quality of life or health-related outcomes. The overall conclusion was that although many older adults reported finding the devices convenient and easier to use than a computer for basic tasks, such as reminders or checking the weather, they sometimes struggled to make themselves understood or to remember the necessary commands. The authors noted the lack of research on effects of use on quality of life, well-being, or functional capacity.

Since then, at least 2 studies have examined socioemotional outcomes of smart device use, although neither compared these devices with text-based devices, such as computers. In a small, randomized trial, 34 older adults were assigned to use either a smart speaker (audio only) or a smart display (audio plus screen) for at least 30 minutes a day for specified activities (eg, listening to music, calling a friend, and playing games) over 12 weeks [19]. Both groups showed significant reductions in loneliness and increases in social support over 12 weeks, with greater changes in the smart display group. In a second, larger but nonrandomized study, 291 older adults living alone were given a smart speaker and training on its use [20]. Participants who were more (vs less) lonely at baseline used it more often over 2 months. Frequent users showed significant decreases in loneliness but not depressive symptoms, whereas intermittent users showed no significant changes in either outcome. In sum, both studies found that frequent use of smart systems was associated with some positive socioemotional changes, although similar changes may have occurred with laptop use or in a control group.

In addition, at least 2 studies have examined health-related outcomes. In the smaller study, 34 adults with type 2 diabetes (mean age 55 years) were assigned to two smart speaker interventions: (1) “standard of care” daily prompts to maintain a log of insulin and blood glucose, or (2) “conversational AI,” in which patients reported their insulin use and blood glucose levels to the smart speaker and received AI-generated dosing instructions based on the data they had just reported [21]. Participants in the conversational AI group achieved optimal dosing in fewer days, were more likely to achieve glycemic improvement and control, and reported greater reductions in diabetes-related emotional distress. The study suggested benefits of AI-generated feedback but did not examine the effectiveness of voice interactivity compared with a text-based system for delivering that feedback.

A larger quasi-experiment [22] examined whether the effects of a smartphone intervention targeting older adults’ depression and health behaviors would be greater if participants also received a smart speaker with additional health-related audio functions (eg, medication reminders, exercise programs, and quizzes). All 170 participants were assigned to use the health app and wearable devices to monitor their health data over 6 months; those who agreed to also receive the smart speaker were compared with those who declined, matched on baseline depression scores. There were no differences between the two nonrandomized groups in changes over time in depression or in 5 of the 6 health behaviors assessed. Only dietary diversity increased more among participants who agreed to receive the smart speaker compared with those who declined.

In sum, despite claims that voice interactivity facilitates accessibility and companionship for older adults, few studies have examined the effects of smart displays or smart speakers on health-related outcomes. Those studies have generally not been large, randomized controlled trials and have seldom compared these devices with text-based platforms. The one quasi-experiment in which older adults chose whether to add a smart speaker to a mobile phone health intervention found minimal evidence of health benefits. As noted earlier, our project is one of the first major trials comparing a smart display with a laptop for delivering an intervention to older adults. It focuses on helping participants manage chronic pain in the context of comorbid health conditions.

### Chronic Pain and Comorbidity

National data from 2023 indicated that 36% of adults aged 65 years and older in the United States experience chronic pain [23]. Chronic pain (ie, lasting more than 3 months) is often accompanied by other chronic conditions, including anxiety and depression [24-26], particularly in older adults [27]. In one study, over 67% of community-dwelling older adults with 2 or more chronic conditions also had chronic pain [28]. Such multimorbidity combined with chronic pain is associated with greater odds of opioid therapy, excessive polypharmacy and medication-related problems, and higher health care expenditures [29-31]. Chronic pain has complex, bidirectional relationships with sleep, exercise, diet, and depression [32-35], all of which affect the management of other chronic conditions. Given the significance of chronic pain for psychosocial quality of life and for management of comorbid conditions [24], we focused on pain interference and psychosocial quality of life as the primary outcomes of a health intervention targeting older adults with multiple chronic conditions.

Various digital therapeutics have been developed to help individuals manage chronic pain. A 2022 meta-analysis of 36 online cognitive and behavioral interventions for adults with chronic pain found small effects on pain intensity, catastrophizing, and interference (*g*=0.27-0.31), with stronger effects observed for interventions that included feedback or guidance from a clinician rather than solely self-paced progress through content [36,37]. Although some of these digital interventions included content targeting comorbid depression and anxiety [38,39], most focused only on pain, often specific to particular conditions such as fibromyalgia, arthritis, or spinal cord injury [40-42]. As such, they were not designed to address the broad range of experiences of older adults with chronic pain and other multimorbid chronic conditions.

In contrast, a recent trial specifically targeted adults with chronic pain and multimorbid chronic conditions, testing the effectiveness of usual care compared with a 6-week program on pain science, self-management, and tailored exercise, involving 2 weekly in-person meetings [43]. Adherence was high: the majority of participants attended more than 75% of the sessions, completed workbook assignments, and reported using the self-management strategies (eg, exercises, breathing, relaxation, and sleep plans). At 12 weeks postintervention, the intervention group showed greater improvements in physical function and greater reductions in various pain-related outcomes compared with the control group. A commentary on the study [44] noted the innovation of focusing on comorbid pain and other chronic health conditions, as well as the possibility of delivering such an intervention remotely, rather than in-person, for longer periods of time.

### This Study

#### Overview

The broad goal was to assess whether delivering a digital eHealth intervention for older adults on a voice-based platform compared with a text-based platform would alter its effectiveness. More specifically, the central question was whether older adults with chronic pain, in the context of multiple comorbid chronic conditions, would show greater use and benefits of an intervention targeting pain and quality of life if they were received it on a smart display rather than a laptop, and whether both the smart display and laptop groups would show benefits compared with a control group that received no device or access to the intervention.

#### Proposed Main Effects

**Figure 1:**
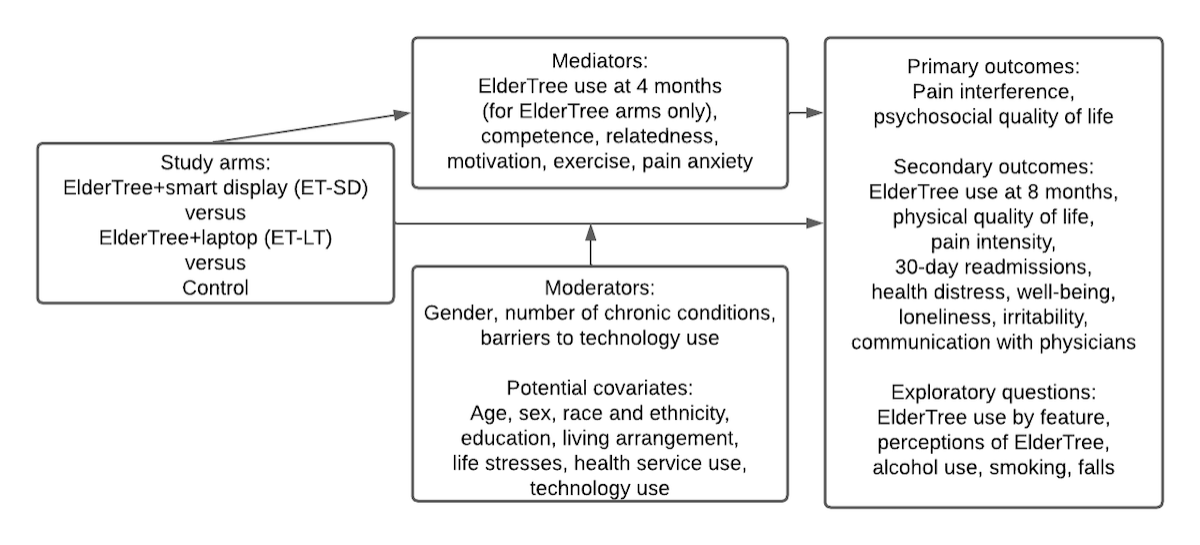
Study logic.

We hypothesized the greatest improvements over the 8-month intervention period in the primary outcomes in the smart display arm, followed by the laptop arm, and then the control group (Figure 1). We also examined whether there would be differences between study arms on the exploratory outcomes. In this study, we focus on the effects of study arm on engagement and the 2 primary outcomes, although we briefly report findings for the other outcomes and provide details in Multimedia Appendix 1.

#### Proposed Mediators

The intervention, called ElderTree, is one of a suite of eHealth systems known as Comprehensive Health Enhancement Support Systems [45,46]. These systems, including ElderTree, are based on principles of Self-Determination Theory, as it is intended to meet 3 core psychological needs related to a particular health situation: competence (self-efficacy to achieve health goals), relatedness (feelings of connection with others dealing with the same health challenges), and intrinsic motivation (ie, feelings of autonomy rather than coercion in working toward health goals) [47]. As shown in the logic diagram, we hypothesized that 4-month levels of these 3 constructs would mediate the effects of study arm on the primary outcomes. Given the focus on pain interference, we also examined rates of exercise and levels of pain anxiety as mediators. For comparisons of the 2 ElderTree arms, we proposed that higher use of ElderTree at 4 months in the smart display arm would mediate stronger effects of the smart display compared with the laptop arm on the primary outcomes.

#### Proposed Moderators

Randomized controlled trials of previous ElderTree platforms, which targeted older adults’ quality of life and management of chronic conditions (but not pain specifically), found moderated effects. In the first study, socioemotional benefits of ElderTree compared with control were stronger for older adults with high rates of primary care use [48]. This pattern was interpreted as indicating stronger effects for those with more complex health conditions, such as multiple chronic conditions. In the second randomized trial, targeting older adults with multiple chronic conditions, socioemotional benefits of ElderTree compared with control were stronger for women than for men [49]. In this study, we examined whether effects in either of the 2 ElderTree arms, compared with control, would again be stronger for women than for men and for those with higher numbers of chronic conditions. Additionally, given the focus on platform, we considered whether the hypothesized benefits of a smart display compared with a laptop would vary by participants’ reports of their barriers to using text-based versus voice-based devices.

#### The Historical Context

There are 3 aspects of the timing of this study that merit presentation up front to contextualize the methods and results. First, we selected and began rapid cycle testing on the laptop and smart display devices in 2020. From the options available at the time, we chose the Google Nest Hub Max. Although smart displays have since evolved, the device we used had some challenges with Wi-Fi connectivity, voice recognition, and the dictation program within the ElderTree app. More details are provided in the Results section. Second, the study period overlapped with the COVID-19 pandemic, as discussed in the section on recruitment and training. Third, Google discontinued “Conversational Actions” as of June 2023. This was a key functionality required to access ElderTree. We stopped recruitment 4 months early and ended the intervention early for 8 smart display participants.

## Methods

### Trial Design

We used a nonblinded, randomized controlled trial design with 1:1:1 allocation to (1) a smart display with internet access and ElderTree, along with usual care; (2) a touchscreen laptop with internet access and ElderTree, along with usual care; or (3) usual care alone. Assessments were conducted at baseline, 4 months, and 8 months.

### Participants

Older adult patients with at least 3 high-risk chronic conditions were recruited from the University of Wisconsin (UW)—Madison Department of Family Medicine and General Internal Medicine system as well as community organizations in the Madison, Milwaukee, and Beloit, Wisconsin, areas. Eligible participants were aged 60 years or older and had chronic pain—indicated by having received a chronic pain diagnosis, by reporting pain on some or most days lasting at least 12 weeks, by reporting pain in the past 3 months on some, most, or all days, and by reporting pain intensity of at least 3 on a scale of 0 (no pain) to 10 (worst imaginable pain) in the past 7 days. They also had at least 3 of the most prevalent chronic conditions among older adults as reported by the Centers for Medicare and Medicaid Services [50]—chronic obstructive pulmonary disease, asthma, diabetes, hyperlipidemia, hypertension, ischemic heart disease, atrial fibrillation, heart failure, stroke, cancer, chronic kidney disease, depression, osteoporosis, and arthritis. We modified the list by adding obesity (BMI≥30) and dizziness/falls/loss of vestibular function. In addition, patients agreed to allow reports about their health tracking to be sent to their primary care provider. Patients were excluded if they required an interpreter or had a medical diagnosis of Alzheimer disease, dementia, schizophrenia or other psychotic disorder, autism spectrum disorder, a known terminal illness with less than 6 months to live, or an acute medical problem requiring immediate hospitalization**.**

### Intervention Groups

Patients in all 3 conditions continued with their usual care provided by primary care and internal medicine clinics in the UW–Madison system and were randomized to one of the following arms.

#### Control

Participants continued with usual care and did not receive a study device, ElderTree, or internet service. Participants were compensated US $30 for each survey completed at baseline, 4 months, and 8 months.

#### ElderTree Laptop

Participants in the laptop arm were given a Google Chromebook with a touchscreen, access to the ElderTree website, and internet access using a hotspot if needed, for 8 months. ElderTree staff conducted a home visit or phone call (if preferred by participant) during which the participant was trained using the laptop, navigating the ElderTree website, and reminded of study expectations: log into ElderTree regularly and explore its features, complete the weekly surveys, and fill out and return the 2 mailed surveys at 4 and 8 months. Participants were compensated US $10 for each mailed outcome survey completed at baseline, 4 months, and 8 months.

#### ElderTree Smart Display

Participants in the smart display arm received a Google Nest Hub Max smart device with a 10-inch touchscreen, access to the ElderTree app, and internet access using a hotspot if needed, for 8 months. Training on using the smart display and ElderTree was provided through a home visit or phone call. Study expectations and compensations were the same as those described for the laptop arm.

#### Laptop

As in earlier iterations of ElderTree, the laptop platform was a members-only website free of advertisements, with design features based on previous users’ feedback and best practices for older populations, such as larger fonts, fewer options, and uncluttered screens for better comprehension, navigation, and usability [51,52]. The site included the following: “discussion groups,” chatroom-like discussions with other participants; “private messages,” an email-like feature for sending and receiving one-to-one messages; “wellness activities,” including journal writing, breathing exercises, guided meditation, and exercise videos designed specifically for older adults; “health library,” reliable online information on topics such as medication, mental and physical health, and talking with a doctor; “weekly survey,” a short questionnaire that asked about general health variables relevant to all chronic conditions, such as sleep, relationship quality, falls, and missed medications; and “living well with chronic pain,” a self-paced course presenting the latest science on chronic pain and strategies for managing it.

#### Smart Display

The smart display platform of ElderTree, developed as a third-party app on the Google Nest Hub Max, was intended to offer the same services as on the laptop platform, although participants navigated by voice and touch. Participants needed to “wake” the device with their voice and say “Hey Google, ElderTree” for the app to open. The device then gave prompts to take the weekly survey or to check out features such as the chronic pain lessons or health library, which included the same video content used on the laptop platform but not the text-based items. These videos were organized as supplemental resources related to each chronic pain lesson. Participants could use voice commands or tap the screen to select features. Because participants sometimes struggled to remember commands, “cheat sheets” with instructions were provided, and a small sticker with the phrase “Hey Google, ElderTree” was placed on the front of the device.

It became clear during development and the course of the study that the smart display dictation function was error-prone, could process only one sentence at a time, and was difficult to correct. Given this, the text-based discussion boards and journaling functions remained available to participants but were hard to use. As described below, a new feature was added to both study arms.

#### Adding the Weekly Meetup

The importance of maintaining social relatedness services was reinforced by the fact that the study took place during the later phases of the COVID-19 pandemic. To address this, we created a synchronous weekly virtual meetup. To maintain the integrity of the research design, meetups were made available to both laptop and smart display participants; they began in August 2022 and continued through the end of the intervention in August 2023. Meetups focused on a new health-centered topic each week (eg, managing chronic pain, balance, memory, and eye health), were facilitated by research staff who provided a summary of the literature on the topic, and were followed by an open discussion. Time was also allocated for participants to share personal joys and sorrows. Each session ended with a group movement exercise and was documented and summarized in an email within a week, along with additional related information.

### Measures

Participants were assessed for all study variables at baseline, 4 months, and 8 months.

#### Primary Outcomes

We assessed the 2 primary outcomes using subscales of the PROMIS-29 (Patient-Reported Outcomes Measurement Information System 29-item profile) Global Health measure [53,54], which has been found to be highly sensitive to changes in pain interference and quality of life [55].

The pain interference subscale included 4 items about the past 7 days (eg, “How much did pain interfere with your day-to-day activities?”). Scoring of individual items varies, and is determined by the scale developers, with higher values indicating greater pain interference.

Psychosocial quality of life was measured using the 12-item subscale from the PROMIS-29, which includes three subcategories: (1) ability to participate in social roles and activities (eg, “I have trouble doing all of my regular leisure activities with others”), (2) anxiety (eg, “I felt fearful”), and (3) depression (eg, “I felt worthless”). Items asked participants to report on the past 7 days. Scoring of individual items varies and is determined by the scale developers, with higher values indicating better quality of life.

#### Mediators

We measured 6 possible mediators. ElderTree use was measured by the number of days of use in the 4-month period before each person’s 4- and 8-month survey completion. Competence was assessed with the 6-item Self-Efficacy for Managing Chronic Disease scale, referring to the past 4 months. Relatedness was assessed with the 5-item McTavish Bonding Scale [56], referring to the past 4 months. Motivation was assessed with 4 items from the Treatment Self-Regulation Questionnaire, referring to the past 4 months [57]. Exercise was measured with a 4-item scale created for this study, referring to the past 4 months. Pain anxiety was assessed with the 13-item Pain Catastrophizing Scale [58], referring to the past 4 months.

#### Moderators

We investigated whether the effects of study arm on change from baseline to endpoint in primary outcomes were moderated by gender, number of chronic conditions, barriers to technology use, and ElderTree use (for laptop vs smart display only).

Gender was reported by participants at baseline (male, female, and self-describe); all participants identified as either male or female.

The number of chronic conditions was reported by participants, who checked all that applied from a list of the most prevalent chronic conditions among older adults, as reported by the Centers for Medicare and Medicaid Services.

Barriers to assigned technology use were reported by participants. Participants in both study arms indicated whether any of the following factors made it difficult for them to use a computer, iPad, or smart speaker (eg, Alexa or Google Home) on a scale from 0 (never used that technology) to 5 (extremely difficult), with 1 representing “not at all”: vision (even with glasses), hearing (even with a hearing aid), voice issues (difficulty speaking clearly), lack of knowledge on how to use the device, memory, and other (please specify).

#### Potential Covariates

Potential covariates included age, gender, race and ethnicity, education, living arrangement, life stressors, unplanned health care use (urgent care or emergency department visits), and comfort with technology use. Participants reported sociodemographic variables including gender, race and ethnicity, education, and whether they lived alone or with others. To assess life stressors, they reported on 14 possible stressors from the Social Readjustment Rating Scale (eg, “Death of a very close friend or family member,” “Change in financial status”) [59], checking all that applied (possible range=0-14). Participants reported unplanned health care use in the preceding 4 months to urgent care or the emergency department. Participants rated their comfort with 6 communication technologies—computer, smartphone, tablet, smart speaker, email, and Facebook—on a 6-point scale (total possible range=0-30), with higher scores indicating greater comfort.

#### Use and Perceptions of ElderTree

To assess system use, time-stamped usage data from ElderTree participants were continuously captured in our database, including specific services used, the date and time the system was accessed, and text entered in discussions and comment threads. Usage data should be interpreted with caution, as quantifying eHealth intervention use is complex and no established method has yet been validated [60].

Use was defined as visiting any service or feature, including the ElderTree home screen. Days of use were calculated as the number of days in which participants accessed ElderTree at least once in a 24-hour period, excluding the training period. Means for this variable included participants with no days of use (scored 0). We also attempted to assess hours of use of ElderTree. Because smart displays did not track time spent on each page, this calculation required estimation and cut-off rules. Calculations and findings for hours of use are presented in Multimedia Appendix 1 to contribute to the emerging discussion on measuring use [60].

#### Attendance and Participation at Meetups

We counted the number of meetup sessions attended by each participant and, for each session, recorded whether they contributed beyond an initial greeting (ie, raised hand and spoke or typed content into the chat).

Perceptions of ElderTree were assessed in the 8-month survey. Participants rated 9 items on a 1-4 scale (1=not at all to 4=very much so) addressing health benefits (eg, “it helped me learn more about my health issues,” “it helped motivate me to take care of my health,” Cronbach α=.80), socioemotional benefits (eg, “I enjoyed the other people on ElderTree,” “it made me realize I am not alone,” Cronbach α=.84), and ease of use (eg, “I found it easy to use,” “it was easy to find what I was looking for,” Cronbach α=.54). Staff also recorded any inquiries or issues reported by participants regarding ElderTree or either device throughout the trial.

### Qualitative Insights

On the 4- and 8-month surveys, participants were asked 3 open-ended questions: “What stopped you or hindered you from using ElderTree or the computer/smart system in general? If you got a computer or smart system from us, what did you like best about getting the computer or smart system in this study? Any other comments or suggestions for us?” We also logged comments about ElderTree on the discussion boards or during phone calls to staff for help with the devices. Comments were unitized (broken into constituent parts) and inductively coded into core themes by the second author. An independent coder categorized a random sample of 25% of unitized comments into the themes (Krippendorff α=.90).

### Sample Size Determination and Power

A 3-point difference (*d*=0.30) in pain interference is considered clinically meaningful [61]. Given this, the study was powered to detect *d*=0.30 between the laptop and smart display groups, and between the laptop and control groups. We ran 10,000 linear mixed model simulations, which indicated that, for a power of >80% to detect a study arm×time interaction of this magnitude, a postattrition sample of 255 participants would be required. Given relatively low rates of attrition and missing data in earlier trials of ElderTree, we recruited a total of 269 participants.

### Recruitment

The UW-Madison Clinical and Health Informatics Institute queried UW Health clinic electronic health records to identify patients who met eligibility criteria. Potential participants received an opt-in letter and consent form from the UW-Madison’s Clinical Trials Institute. The letter described the study and included a postage-paid return invitation for further contact from the study team.

To supplement UW Health recruitment and diversify the patient population, we worked with Access Community Health Centers in Madison, Wisconsin, and community collaborators with strong ties to local African American health care and patient communities. Staff at Access Community Health Centers identified potentially eligible patients and distributed the recruitment flyer, consent form, and return invitation. Community collaborators promoted the study opportunity within Black churches, community centers, fraternity and sorority health services programs, and other organizations in the Madison and Beloit, Wisconsin, areas.

The recruitment period (July 2021-December 2022) overlapped with the COVID-19 pandemic. Because the primary mode of recruitment was opt-in letters and participants could choose no-contact phone training rather than in person training, accrual proceeded on schedule. Supplemental community recruitment efforts focused mainly on flyer distribution, email newsletters, and other contact-free strategies.

### Randomization and Training

The project manager used a computer-generated allocation sequence to randomize patients on a 1:1:1 ratio to ElderTree Smart Display (ET-SD), ElderTree Laptop (ET-LT), or control group, stratified by gender (male, female), site (UW Health, Access Community Health Centers, community), and number of chronic conditions (≤5 or ≥6). Block size was 12. When baseline assessment and consent were complete, research staff opened the sealed opaque envelope revealing group assignment and conducted equipment setup and training for the ET-SD and ET-LT arms, including all services and instructions for use). Training was conducted at the participants’ homes or a public location (eg, public library), unless participants preferred no-contact training due to COVID-19 or other reasons, in which case equipment was mailed and training occurred by phone. No training was provided for the control arm. Once assigned, participants could not be blinded to condition, as those in the ET-SD and ET-LT arms were asked to use ElderTree while control participants were not. The training researcher also could not be blinded to condition after assignment was revealed.

### Statistical Methods

Following randomization, the 3 groups were assessed for comparability across all prespecified stratification variables. No statistically significant differences were found between arms for gender, site, or number of chronic conditions.

We selected covariates by examining whether they were moderately correlated (*r*≥0.30, averaged across the 3 time points) with at least one of the primary outcomes [62]. Race and ethnicity were dichotomized (non-Hispanic White vs all other groups) and were unrelated to either outcome. Only life stressors met the correlation threshold (pain interference *r*=0.35; psychosocial PROMIS-29 *r*=−0.46) and was retained as a covariate.

Normality, linearity, and homogeneity of variance for outcome data were assessed using descriptive statistics and graphical representations. Outcomes were analyzed with mixed-effects models using the “glmer()” function from the *lme4* package in R (R Foundation for Statistical Computing. These models account for correlated measurements within participants, incorporated all available data (allowing for intention-to-treat rather than complete-case analysis), and provide unbiased estimates when data were missing at random. Each model included a random effect for participant and fixed effects for survey time point, study arm, and arm-by–time point interaction. Survey time point was entered as a continuous variable with 3 values (baseline, 4 months, and 8 months).

### Ethical Considerations

This study was approved by the UW Health Sciences and Minimal Risk Research Institutional Review Board (reference number 2020-0868) and registered at ClinicalTrials.gov (NCT04798196). Verbal informed consent was obtained by research staff during the initial recruitment call. A unique “study ID” was assigned at enrollment and associated with all data for the duration of the study to protect participant privacy and confidentiality. Participants received compensation (US $30) for completing surveys at baseline, 4 months, and 8 months.

## Results

### Participants

**Figure 2:**
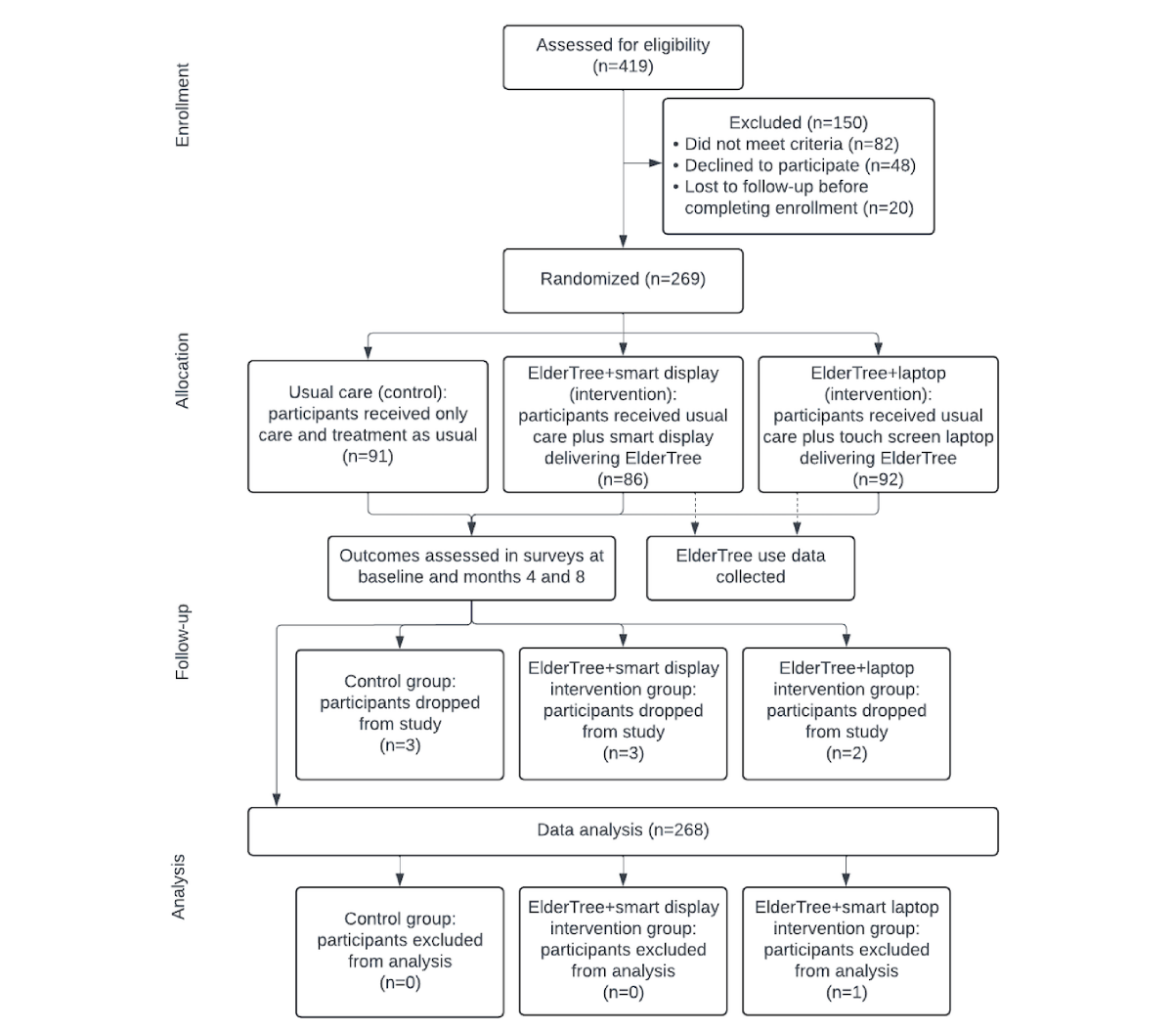
Consolidated Standards of Reporting Trials (CONSORT) flow diagram. Data were analyzed using mixed-effects models, which incorporate all available data rather than only complete cases.

A total of 269 participants were randomized (91 to control, 92 to ET-LT, and 86 to ET-SD); one participant was excluded because they did not complete the baseline survey, leaving 268 participants for analysis (Figure 2). Recruitment began in July 2021 and ended in December 2022; the intervention period continued through August 2023, which coincided with the historical context described above. Eight ET-SD participants were affected by Google’s decision to discontinue the functionality that enabled access to ElderTree on the smart display: their intervention periods were 2 weeks to 2 months shorter, depending on enrollment date.

Participant baseline characteristics are reported by study arm in Table 1. Across all participants, 177 (66%) identified as female, and 211 (78.7%) identified as White only. The mean age was 69.8 (SD 7.34) years, and number of chronic conditions was 6.54 (SD 2.46).

**Table 1:** Participant characteristics by study arm at baseline.

Characteristic			Control (N=91)		ET-LT^a^ (N=91)		ET-SD^b^ (N=86)
**Gender, n (%)**							
	Female	63 (69)		59 (65)		55 (64)	
	Male	28 (31)		32 (35)		31 (36)	
**Race, n (%)**							
	Asian only	0 (0)		1 (1)		0 (0)	
	Black or African American and American Indian or Alaskan Native	2 (2)		1 (1)		0 (0)	
	Black, Euro-African, or African American	20 (22)		16 (18)		14 (16)	
	White only	68 (75)		72 (79)		71 (83)	
	White and Hispanic or Latino	1 (1)		1 (1)		0 (0)	
	White, Black or African American, American Indian or Alaskan Native	0 (0)		0 (0)		1 (1)	
**Highest level of education, n (%)**							
	Elementary school	3 (3)		0 (0)		0 (0)	
	High school	12 (13)		17 (19)		12 (14)	
	Some college	19 (21)		18 (20)		19 (22)	
	Vocational or technical school	16 (18)		13 (14)		11 (13)	
	College graduate	24 (26)		25 (27)		25 (29)	
	Postgraduate or professional	16 (18)		18 (20)		19 (22)	
**Living situation, n (%)**							
	Had significant other	50 (55)		49 (54)		52 (60)	
	Live alone	52 (57)		55 (60)		53 (62)	
Had unplanned health service use, n (%)			18 (20)		21 (23)		20 (23)
Age (years), mean (SD)			69.49 (7.77)		69.76 (7.39)		70.13 (6.86)
Number of chronic conditions, mean (SD)			6.52 (2.31)		6.54 (2.54)		6.57 (2.55)
Life stressors (range 0-14), mean (SD)			2.81 (2.29)		2.69 (1.83)		2.65 (2.27)

^a^ET-LT: ElderTree laptop.

^b^ET-SD: ElderTree smart display.

#### Effects of Study Arm on Changes Over Time in Primary Outcomes

The central questions of this study were (1) whether delivering the intervention on different platforms affected changes over time in the primary outcomes of pain interference and psychosocial quality of life, and (2) whether both intervention arms would outperform the control group.

#### Pain Interference

Visualizations of the data indicated that, descriptively, the 3 study arms differed in baseline levels of pain interference (despite randomization), with different patterns by gender. Before examining the effects of study arm on changes over time, we first examined whether these baseline differences were significant. The results of 2-way ANOVAs predicting baseline pain interference found no significant effects of study arm, gender, or the gender×study arm interaction, comparing all 3 study arms (Table S1 in Multimedia Appendix 1). The analyses described below focus on changes over time in pain interference (ie, controlling for baseline scores), so initial differences would have been incorporated. Nonetheless, it is useful to indicate that the groups were statistically comparable at baseline despite descriptively different levels.

We did not find a significant difference between ET-LT and ET-SD for changes in pain interference over time (arm×time interaction b=–0.11, 95% CI –1.07 to 0.85; *P*=.82). There was also a nonsignificant difference between the combined ET-LT+ET-SD groups and control for change in pain interference over time (arm×time interaction b=–0.41, 95% CI –1.23 to 0.41; *P*=.33).

#### Psychosocial Quality of Life

Visualizations of the data indicated that, descriptively, the 3 study arms differed in baseline levels of psychosocial quality of life (despite randomization), with different patterns by gender. Again, we began by examining whether these differences were significant: they were not. Two-way ANOVAs predicting baseline psychosocial quality of life found no significant differences by study arm (comparing all 3 arms or ET-LT+ET-SD vs control), nor significant interactions between study arm and gender. However, women scored significantly higher than men (Table S1 in Multimedia Appendix 1).

There was no significant difference between ET-LT and ET-SD for change in psychosocial quality of life over time (arm×time interaction b=–0.21, 95% CI –0.96 to 0.55; *P*=.56). There was also a nonsignificant difference between the combined ET-LT+ET-SD groups and control in change in psychosocial quality of life over time (arm×time interaction b=0.04, 95% CI –0.61 to 0.69; *P*=.90).

#### Mediation of Effects of Study Arm

No mediation analyses were conducted on the primary outcomes because the effects of study arm on these were nonsignificant.

#### Moderation of Effects of Study Arm

We examined whether the effects of study arm on changes over time in the primary outcomes were moderated by gender, number of chronic conditions, and barriers to technology use.

#### Gender and Changes in Pain Interference

**Figure 3:**
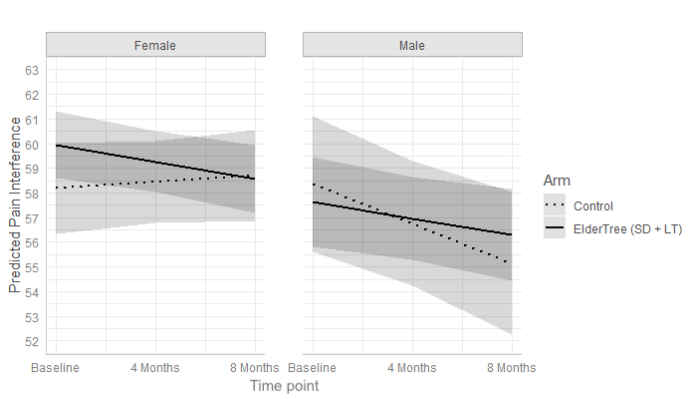
Predicted mean pain interference scores over time, stratified by gender.

Gender did not moderate the effect of ET-LT versus ET-SD on changes over time in pain interference (b=–1.56, 95% CI –3.56 to 0.44; *P*=.13). However, as illustrated in Figure 3, gender did moderate the effect of the combined ET-LT+ET-SD groups versus control (b=1.91, 95% CI 0.11-3.71; *P*=.04). For women (n=177), those in the combined ET-LT+ET-SD group showed a significant decrease in pain interference over time (b=–0.69, 95% CI –1.29 to –0.10; *P*=.02) but those in the control group showed no significant change (b=0.25, 95% CI –0.53 to 1.04; *P*=.53). For men (n=91), those in the combined ET-LT+ET-SD group showed a nonsignificant decrease (b=–0.67, 95% CI –1.47 to 0.14; *P*=.10); those in the control group showed a significant decrease (b=–1.61, 95% CI –2.88 to –0.35; *P*=.01). However, even the significant decreases did not meet the benchmark for meaningful reductions. At 8 months, (controlling for baseline scores) there were no significant differences between study arms in pain interference for women (*P*=.10) or men (*P*=.27).

#### Gender and Changes in Psychosocial Quality of Life

**Figure 4:**
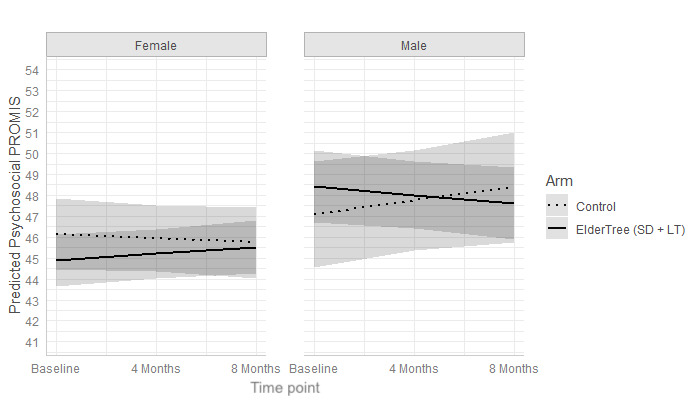
Predicted mean psychosocial quality of life scores over time, stratified by gender.

Gender did not moderate the effect of ET-LT versus ET-SD on changes over time in psychosocial quality of life (b=1.27, 95% CI –0.31 to 2.85; *P*=.12). However, as illustrated in Figure 4, gender did moderate the effect of the combined ET-LT+ET-SD group versus control (b=–1.59, 95% CI –3.01 to –0.17; *P*=.03). For women, neither group showed significant changes over time: ET-LT+ET-SD (b=0.32, 95% CI –0.15 to 0.79; *P*=.18) and control (b=–0.21, 95% CI –0.36 to 1.64; *P*=.21). For men, the ET-LT+ET-SD group showed a significant decrease in psychosocial quality of life over time (b=–0.69, 95% CI –1.29 to –0.10; *P*=.02) whereas the control group remained stable (b=0.64, 95% CI –0.36 to 1.64; *P*=.21). None of the changes reached the benchmark for meaningful differences. At 8 months (controlling for baseline scores), there were no significant differences between study arms (control vs ET-LT vs ET-SD) for women (*P*=.25) or men (*P*=.13).

#### Other Moderators

To preview briefly, no additional moderation effects were observed. Number of chronic conditions did not moderate the effect of ET-LT versus ET-SD on changes over time in pain interference (b=–0.16, 95% CI –0.63 to 0.31; *P*=.51) or psychosocial quality of life (b=–0.32, 95% CI –0.70 to 0.06; *P*=.10). Nor did it moderate the effect of the combined ET-LT+ET-SD group versus control on changes over time in pain interference (b=–0.01, 95% CI –0.39 to 0.36; *P*=.94) or psychosocial quality of life (b=0.24, 95% CI –0.06 to 0.54; *P*=.12).

Barriers to technology did not moderate the effect of ET-LT versus ET-SD on changes over time in pain interference (b=0.97, 95% CI –1.20 to 3.15; *P*=.38) or psychosocial quality of life. Nor did they moderate the effect of the combined ET-LT+ET-SD group versus control on changes over time in pain interference (b=–0.55, 95% CI –2.50 to 1.40; *P*=.58) or psychosocial quality of life (b=0.28, 95% CI –1.27 to 1.84; *P*=.72).

#### Summary of Effects of Study Arm on Secondary and Exploratory Outcomes

As shown in Table S2 in Multimedia Appendix 1, there were no significant differences between ET-LT and ET-SD, or between the combined ET-LT+ET-SD groups versus control, in changes over time for any other secondary outcomes: physical quality of life, pain intensity, 30-day hospital readmissions, health distress, well-being, loneliness, irritability, or communication with physicians.

As shown in Table S3 in Multimedia Appendix 1, there were also no differences for either set of comparisons in changes over time for the exploratory outcomes of alcohol use, cigarette use, and falls.

### ElderTree Usage and Ratings Overall and by Platform

#### Usage

Overall, the weekly survey was the most used service for both ET-LT and ET-SD. Weekly reminders by email and text prompted participants to take the survey; it took only a few minutes to complete and was easy to navigate on both platforms.

Other use patterns varied by platform. As shown in Table 2, participants assigned to ET-LT (vs ET-SD) used ElderTree on more days (Figure S1 in Multimedia Appendix 1 provides estimates of hours of use). In months 1-4, ET-LT participants used Elder Tree a mean of 28.47 (SD 19.28) days, compared with ET-SD participants, who used it for a mean of 19.02 (SD 16.09) days. In months 5-8, ET-LT participants used it a mean of 17.48 (SD 14.34) days, compared with ET-SD participants, who used it a mean of 10.08 (SD 9.85) days. These differences (approximately 1 week of use in each 4-month period) were significant (incidence rate ratio [IRR] 0.60, 95% CI 0.48-0.75; *P*<.001) and did not change in magnitude over time (IRR 0.85, 95% CI 0.71-1.02; *P*=.08).

**Table 2:** Laptop versus smart display in use and perceptions of ElderTree.

Outcome		Laptop (n=91), mean (SD)	Smart display (n=86), mean (SD)	Main effect of arm				Arm×time interaction		
				Estimate	95% CI	*P* value	Estimate		95% CI	*P* value
**Days of ElderTree use**										
	Pain modules	4.95 (5.26)	4.92 (8.00)	0.97^a^	0.68 to 1.38	.86	1.25^a^		0.84 to 1.86	.27
	Discussion group	12.36 (14.4)	2.39 (3.37)	0.18^a^	0.13 to 0.26	<.001	0.58^a^		0.38 to 0.87	.009
	Health library	4.20 (5.50)	0.48 (0.89)	0.07^a^	0.04 to 0.12	<.001	0.29^a^		0.12 to 0.74	.01
	weekly Survey	14.57 (6.89)	10.18 (6.06)	–4.32^b^	–6.12 to –2.52	<.001	0.56^b^		–1.12 to 2.23	.51
**Perception of ElderTree**										
	ElderTree overall	3.02 0.58	2.59 0.65	–0.46^b^	–0.66 to –0.25	<.001	0.05^b^		–0.10 to 0.19	.52
	Health benefits	2.72 0.74	2.44 0.78	–0.33^b^	–0.59 to –0.08	.01	0.08^b^		–0.12 to 0.27	.44
	Socioemotional benefits	2.91 0.79	2.48 0.93	–0.51^b^	–0.80 to –0.21	<.001	0.13^b^		–0.09 to 0.35	.25
	Ease of use	3.42 0.53	2.86 0.70	–0.55^b^	–0.77 to –0.34	<.001	–0.04^b^		–0.24 to 0.15	.66

^a^IRR: incidence rate ratio (for count outcomes).

^b^Regression coefficient (for continuous outcomes).

In terms of participation in the weekly meetups, among participants who remained in the study after meetups were offered, 39% (28/74) of ET-LT users and 36% (20/64) of ET-SD users attended at least once. In total, 48 participants (out of 138 possible) attended a meetup at least once (34.8%), and many attended multiple times resulting in 639 meetup admissions. Of those who participated each week, an average 70% (441/639) were ET-LT users and 30% (198/639) were ET-SD users. Only 6 (out of 28) ET-LT users and 4 (out of 20) ET-SD users attended but never contributed to the discussion during the meetup beyond an initial greeting. Participants were encouraged to participate, but it was not required if they felt more comfortable just listening.

There was no difference between ET-LT and ET-SD in the number of chronic pain modules started (overall mean 3.16 out of 8, SD 2.76), but more modules were completed in the ET-LT group (mean 2.63, SD 3.19) than in the ET-SD group (mean 1.74, SD 2.68; *P*=.04). There were no differences between ET-LT and ET-SD groups in the likelihood of attending one of the meetups (odds ratio [OR] 0.87, 95% CI 0.43-1.75; *P*=.71) or contributing verbally at a meetup (OR 0.92, 95% CI 0.43-1.95; *P*=.83).

#### Ratings of ElderTree

As shown in Table 2, mean ratings for benefits of ElderTree overall, health benefits, socioemotional benefits, and ease of use were generally above the mid-point (1=not at all to 4=very much). As Table 2 also shows, those in the ET-LT (vs ET-SD) group gave significantly higher ratings (averaged across the 4- and 8-month surveys) for all 4 measures. Significant arm×time effects are illustrated in Figures S2 and S3 in Multimedia Appendix 1.

#### Effects of ElderTree Usage and Perceptions on Primary Outcomes

Given that we did not find significant overall study arm effects on our primary outcomes, we examined whether ElderTree usage and perceptions predicted change in our primary outcomes. We calculated change scores for our primary outcomes by subtracting baseline scores for each outcome from the mean of that outcome across the 4- and 8-month assessments. We regressed these change scores for primary outcomes on the total number of days of ElderTree use and mean perceptions in separate models for each predictor and outcome. We also estimated separate models using either a combined sample with both ElderTree platforms (ET-LT+ET-SD) or separate samples for ET-LT and ET-SD. We did not apply any correction to *P* values to address α inflation due to multiple testing. Neither ElderTree usage nor any of the ElderTree perception scales significantly predicted change in primary outcomes across these 30 exploratory analyses (ie, 2 outcomes×5 predictors×3 samples; *P*=.10-.96).

#### Probing Possible Gender Differences in Uses

Although gender only moderated the effect of the combined ET-LT+ET-SD versus control, we explored whether there were gender differences in uses of ElderTree on the laptop versus smart display. We averaged across the entire study period for each person to simplify the models because models with time included produced convergence errors. Gender did not alter the differences between ET-LT and ET-SD for days of use (b=–0.21, 95% CI 0.23-0.94; *P*=.34), hours of use (b=–0.42, 95% CI 0.29-1.43; *P*=.15), likelihood of attending a meetup (arm×gender OR 0.90, 95% CI 0.18-4.27; *P*=.89), or talking at a meetup (arm×gender OR 0.81, 95% CI 0.14-4.36; *P*=.81).

#### Qualitative Insights

Textbox 1 lists core themes and example comments about perceived benefits of ElderTree. Health-related comments referenced the value of the breathing and exercise videos, increased awareness and knowledge of their conditions, and learning from the pain modules. Participants also commented on social connections, including the benefits of connecting with others facing similar challenges. Several participants wrote positively about platform affordances, with smart display users discussing the benefits of voice access and laptop users expressing a preference or interest in typing.

**Participant comments about perceived benefits: themes and examples. Codes are anonymized identifiers. Comments could reflect multiple themes and were categorized accordingly. LT: laptop user; SD: smart display user.**

**Health-related uses and benefits of ElderTree**

I liked the fact that it answered a lot of my questions easily and I have access to information and the breathing techniques came in real handy.

SD AC53

I learned so much on how to control and manage my pain!

SD AC65

I now feel more in control of my physical problems and I'm more willing to try things I've learned in this study.

SD CO14

I like the Weekly Survey because it makes me record my blood pressure readings daily and keep myself well hydrated daily! Plus keep a handle on my weight!

LT AC70

I liked the 8 lessons I learned a lot and picked up some breathing exercises that helped my breathing.... lost 1/2 lung to cancer 10 years ago.

LT EC67

Even when I dont open the computer I do the breathing exercises, lift lite weights, and stand on one leg. I plan to expand on this. I feel better and lost 6 lbs. What can be better than that. This program helped me achieve that…. feel so much better and healthier.

LT YA10

**Social connections**

I really enjoy the Tuesday meet ups. I don't do much of interest so listen but it helps me feel involved.

SD EC86

I brought my report to my doctor and she said “you seem so much calmer…why do you think that may be?” I think it’s because I’m in this study where I’m learning and seeing that I’m not alone… there are others experiencing similar issues related to aging.

LT EC67

I appreciate being able to talk to “real people” without needing to be the “perfect” senior.

LT HB11

**Other features and services of ElderTree**

The games were a welcome diplatform sometimes.

SD CO14

I liked… recipes, and playing some of the games. I also enjoyed doing some journaling.

LT EC15

**Platform benefits**

What I like best about smart system is that I got to talk to it and it talk back. I was excited that it knew my name.

SD YA06

I liked to voice command because the worst of all my chronic pain is in my hands!

SD EC44

Helps me learn how to type

LT AC66

**Other benefits of device**

Could access weather, news, music, podcasts, etc.

SD AC14

Beautiful photos and music

SD AC16

I can look up a lot of info to help myself get motivated. That I can keep in touch with doctors.

LT AC66

The laptop is so convenient to take on our trips…i use it everywhere. in the condo, motels and again at home.

LT YA10

Textbox 2 lists the core challenges and barriers referenced by participants. Chief among them were technical difficulties, including Wi-Fi connectivity issues and glitches, as well as limitations and slowness of the dictation function on the smart display. Relatedly, several participants wrote that they preferred their other devices (eg, phone or laptop) or felt too unfamiliar with their assigned device to use it often or effectively. Specific to ElderTree, some participants reported finding the material too easy or feeling that they were too young and healthy for the intervention and could not relate to other participants. Additional barriers included poor health and lack of time or motivation.

**Participant comments about challenges: themes and examples. Codes are anonymized identifiers. Comments could reflect multiple themes and were categorized accordingly. LT: laptop user; SD: smart display user.**

**Technical issues: Wi-Fi connectivity and ElderTree glitches**

Every time (yes, every time) I used the ElderTree smart system, it couldn't stay connected to our Spectrum internet. Same problem when it was connected to ElderTree/Google's internet. Had to constantly reboot and/or just give up for that day. I was never able to connect to meetings with other participants.

SD CO14

Lessons seemed difficult to complete in one session. Would be interrupted by “glitch” ie 2 pain lessons had to be completed in 3 or 4 attempts often resulting in loss of interest.

SD EC25

Was very disappointed in the Journaling feature – too slow – made it useless for me.

SD YA03

Google smart device – the dictation system was useless.

SD EC175

For some reason, computer runs very slow. Going back to read next, notice it seems to lock up. Sometimes I need to log out and back in to get it to work. Then I just don't use it

LT YA46

**Unsure how to use device or navigate ElderTrees**

I simply am not able to manage the device. Family members would respond with “Wow- you've got a...(thing)... began trying it out - I could not follow their enthusiastic directions- and any possible lesson was gone in a flash. I would call for help from the Tech Support number often. (I loved the pictures and time+temp).

SD EC84

I am not very computer savvy. Took me awhile to navigate the various offerings (classes, library, etc).

LT EC67

**Did not like device (prefer other devices)**

I am used to using a computer a lot. If I accessed ElderTree from a laptop rather than from the Google device I would have used it a lot more because I am often on the computer. But, I would need to make a special effort to go on to the Google device just for ElderTree

SD YA68

Have the smart speaker. Don't like that I can't turn off the camera and audio separately, so I keep it turned off.

SD EC33

**Content pitched at wrong level, community not ideal fit**

Sessions were very slow. Exercises very basic. Would have been better to offer levels of difficulty.

LT EC105

Consider grouping participants. It’s hard for me to relate to folks socially who live in care communities.

LT EC151

**Health issues**

I have trouble with tremors, recently saw a neurologist and was told I am showing signs of Parkinsonisms.

LT EC194

I had bronchitis and was too sick to use it for several weeks

SD HBO5

**Too busy with other things, under-motivated**

I had other things, like reading books for 3 book groups I am in.

LT EC61

I was often busy (taking a class, traveling, having guests, working on non-health life issues) and didn't spend consistent time on ElderTree. This interfered or slowed down my ability to make habit changes that ElderTree helped me to identify that were needed. Still working on these.

LT EC114

## Discussion

### Overview

The goal of this large-sample, 8-month randomized controlled trial was to test the hypothesis that a smart display would be even better than a laptop for delivering a digital health intervention to older adults with chronic pain and multiple other chronic conditions. By “even better,” we meant that participants randomized to use the intervention on the smart display, rather than the laptop, would use it more and show greater improvements over time in health-related outcomes. “Even better” also involved a comparison with the control group: older adults in both intervention arms were expected to show greater improvements over time relative to those receiving treatment as usual. As noted earlier, this appears to be one of the first large, randomized controlled trials to assess these comparisons, rather than focusing solely on feasibility and use. In discussing the findings, we first address the health-related outcomes before examining the use data and lessons learned.

First, in the sample as a whole, there were no significant differences between the two ElderTree platforms (laptop vs smart display) with respect to changes over time in any of the primary, secondary, or exploratory health-related outcomes. Second, there were no significant differences between the combined ElderTree group (laptop+smart display) and the control group on changes over time in any of the primary, secondary, or exploratory outcomes. Moreover, exploratory analyses did not detect any relationship between days of use or perceptions of ElderTree (whether combined across platforms or separately) and change in either primary outcomes. In other words, participants who used ElderTree more frequently or reported more positive perceptions of either platform did not show greater improvement in pain interference or psychosocial quality of life. However, our coarse measure of total days of use may have been too coarse to detect meaningful differences based on usage.

The moderation analyses found no differences between ET-LT and ET-SD on any health-related outcomes for any of the hypothesized subgroups, including participants who reported few barriers to using their assigned platform. Although it was intuitive to expect that laptops would be superior for participants who found it easiest to type and read, and that smart displays would be superior for those who found it easiest to dictate and listen, we did not observe these patterns, even though a few participants mentioned appreciating their particular platform for precisely these reasons (Textbox 1).

For the comparison of the combined ElderTree group (ET-LT+ET-SD) versus control, only gender—not number of chronic conditions or reported barriers to use—was a significant moderator. Although the moderation results were somewhat complex, the overall takeaway is straightforward. Women showed the sole positive effect of ElderTree in the combined group, with a significant reduction in pain interference over time. Men showed the sole negative effect, with a significant decrease in psychosocial quality of life.

We found a similar gender difference in a previous trial of an earlier ElderTree version (focused on multiple chronic conditions), in which women—but not men—showed significant benefits of ElderTree versus an attention control on psychosocial quality of life and psychological well-being [49]. Given meta-analytic evidence that women tend to give and receive more social support online than men [63] we considered whether women in both studies engaged more with the discussion boards (and, in this study, with the meetups). In fact, in both studies, there were no differences between men and women in the amount or type of ElderTree use. Another possibility is that women benefited from the fact that there were more female than male participants in both studies (reflecting the demographics of this age group), so they likely experienced more same-sex interactions on ElderTree than men. Notably, however, when we offered same-sex discussion groups in the first iteration of ElderTree, participants made little use of them, instead converging on the shared discussion groups. Ultimately, we are reluctant to overinterpret the current gender differences, given that neither of the significant changes reached benchmarks for meaningful effects and that there were no significant differences between men and women by the end of the trial. We interpret the current findings as indicating that neither men nor women showed a meaningful effect of ElderTree, but future interventions should engage more men (as well as women) during development phase and in qualitative interviews to gain deeper insights into possible differences.

Why was our pain-focused intervention largely ineffective? As described in the Introduction, previous online interventions for chronic pain have typically found small effects unless participants’ progress was guided by a clinician [38,39]. Our intervention was not clinician-guided, given the goal of creating a readily scalable program, and unlike previous online interventions for chronic pain, we targeted older adults concurrently managing multiple other chronic conditions. We hypothesized that ElderTree’s combination of educational modules, discussion board and meetups, and other resources (eg, meditation and exercise videos, health library, and journaling) would yield positive results, particularly if offered on smart displays that could facilitate voice-activated engagement. We can identify at least 3 reasons for the overall lack of effects, in addition to the absence of clinician guidance and the complex health challenges of our older adult participants.

First, although we tried to make the modules engaging (eg, by adding inclusive images and short quizzes to highlight key points), the static slides with audio format and length (13-22 minutes), combined with some participants’ difficulties with Wi-Fi connectivity and device glitches (Textbox 2), may have contributed to low use of that content. Second, use and effects of ElderTree may have been diminished by the context of the COVID-19 pandemic, not only because of the amount and urgency of other health-related information at the time, but also because, as one participant noted, there was a hunger for in-person rather than online interactions. Third, although the PROMIS-29 and its subscales have been validated, it is possible that the measures of pain interference (4 items) and psychosocial quality of life (12 items) were not subtle enough to assess change in these outcomes. Indeed, the reliance on self-report measures rather than clinical assessments is an important limitation of this study, though, given our primary focus on pain interference and quality of life, this seemed appropriate. Future studies may benefit from other measures, such as mobility and physical function.

Why were days of use and ratings of ElderTree higher in the laptop arm rather than in the smart display arm? We selected the Google Nest Hub Max because its Google Assistant–powered speech recognition, robust developer tools (Google Action SDK), and built-in camera seemed ideal for a voice‐first, multimedia intervention. In practice, however, core ElderTree features did not translate well. Threaded discussion, a core service of ElderTree that thrived on the laptop, became cumbersome on the smart display. Voice dictation forced users into short entries with repeated rerecording, so discussion‐group participation plummeted. Likewise, long‐form readings could not be scrolled or linked through URLs because the device was not designed for text‐heavy content.

The platform of smart display deployed in this study relied on pre-LLM (large language model) dialogue technology. These earlier systems had limited natural language capabilities, required a wake word, and were difficult to navigate, especially for users unfamiliar with command-based input. Additionally, some participants were more familiar with laptops or other devices like iPads or their phones than with smart displays.

We are currently concluding another randomized controlled trial of a subsequent iteration of ElderTree (ET-Move, focused on facilitating mobility in older adults with multiple chronic conditions), also comparing laptops and smart displays as platforms [64]. Based on the lessons of this study, we made several changes. First, to boost engagement and leverage the capabilities of the smart display, we shifted from text-based content to videos. Both the smart display and laptop platforms offer many informational and movement videos. Most are under 5 minutes in length, and, except for the required safety videos, can be accessed in any order preferred by the user. Second, we shifted the emphasis away from pain to the gains achievable from safe exercise. Third, as suggested by some users in this study, we introduced different levels of content; ET-Move offers different types of exercise based on assessments of participants’ physical capabilities. Fourth, we incorporated meetups throughout the study, to serve in part as a proxy for guidance. During these meetups, participants and moderators perform a simple set of exercises together, either before or after the social and educational aspects of the session.

We can see additional avenues for improving future interventions offered on smart displays. Most notably, interventions should incorporate LLM-based voice agents with natural, context-aware, and flexible dialogue. Such systems could support open-ended interaction and reduce the cognitive demands on users by eliminating the need for memorized commands. As AI models continue to evolve, so does the evidence base for their use in health interventions. A systematic review of 21 randomized controlled trials of AI-driven chatbot health interventions (thus far text-based rather than voice-driven on smart displays) found varied impacts on physical activity, functional abilities, adoption of healthier lifestyle habits, understanding of their medical conditions, and improvements in mental and psychosocial well-being [65]. A meta-analysis of 32 studies involving AI conversational agent interventions (also primarily text-based) showed statistically significant short-term improvements across a range of mental health outcomes, although long-term effects were generally nonsignificant [66]. Empathic responsiveness, extended interaction duration, and personal tailoring emerged as key factors enhancing efficacy.

Indeed, a core lesson of this study was that future systems should be tailored to user characteristics such as digital literacy and comfort with technology. Ultimately, while a randomized controlled trial examining platform or modality effects requires participants to use the assigned device, the real-world use of health interventions is likely enhanced by allowing access on the devices most familiar and useful to the participants. Moreover, for older adults who struggle to use the necessary technologies, extended onboarding periods and repeated, multimodal support are needed.

Overall, the results point to the challenges of using an online system to address chronic pain in older adults with multiple chronic conditions, particularly amid the challenges and stresses of a global pandemic. Despite the current findings, it is premature to declare that smart displays offer no benefit over laptops, especially given rapid advances in AI-based capabilities.

### Conclusion

We found little evidence of meaningful effects of our online pain intervention for older adults with multiple chronic conditions, whether delivered on a voice-interactive smart display or on a laptop.

## Appendix

Multimedia Appendix 1 Additional information for outcome analyses.

Multimedia Appendix 2 CONSORT eHEALTH checklist V (1.6.1).

## Abbreviations

AI: artificial intelligence
ET-LT: ElderTree laptop
ET-SD: ElderTree smart display
IRR: incidence rate ratio
LLM: large language model
OR: odds ratio
PROMIS-29: Patient-Reported Outcomes Measurement Information System 29-item profile
UW: University of Wisconsin

## Data Availability

Following the completion of the final analysis, the datasets generated or analyzed during this study will be available from the corresponding author upon reasonable request.
