# Supplementary material for: Using Smart Displays to Implement an eHealth System for Older Adults With Multiple Chronic Conditions: Randomized Controlled Trial
Source: JMIR Aging. 2025 Nov 18;8:e75991. doi: 10.2196/75991 (PMC12673301; doi:10.2196/75991)
Supplement: Multimedia Appendix 1 [file aging_v8i1e75991_app1.docx]

**Appendix. Supplemental Information for Outcome Analyses**

**Calculation of hours of use.** Hours spent on the system was calculated as follows: first the time (in seconds) was calculated between each recorded user action (page click for laptop; voice or click command for smart display) by participant. If the timespan between actions was larger than 41 minutes (the maximum length of the longest video), the time spent for that action was removed. Next, all actions preceding logging into the system were removed. If the seconds spent on the home page or thought of the day exceeded one minute, this time was reduced to 60 seconds. Finally, all actions longer than 10 minutes that were not the following services were reduced to 60 seconds: chronic pain modules, discussion group, health library, private messages, weekly survey, and wellness activities. Time spent in seconds was then totaled for the two study time periods (baseline to 4 months and 4 months to 8 months) by participant and converted to hours. These rules are by no means exhaustive, so some inaccuracy in our time spent calculations may exist and the results for hours of use should be interpreted with caution.

**Table S1.** Baseline pain interference and psychosocial quality of life: Two-Way ANOVAs for Arm (Control vs. Laptop vs. Smart Display) x Gender (Women vs. Men).

|  | Baseline Pain Interference | | |
| --- | --- | --- | --- |
|  | df | F value | P-value |
| Arm | 2 | 0.49 | .61 |
| gender | 1 | 2.78 | .10 |
| Arm x gender | 2 | 1.26 | .28 |
| Residuals | 262 |  |  |
|  | Baseline Psychosocial Quality of Life | | |
|  |  |  |  |
| Arm | 2 | 0.73 | .48 |
| gender | 1 | 6.22 | **.013** |
| Arm x gender | 2 | 1.93 | .15 |
| Residuals | 261 |  |  |

**Note.**  For baseline psychosocial quality of life: women (M = 45.23, SD = 7.62, n = 177) scored lower than men (M = 47.66, SD = 7.55, n = 91), F (1, 357) = 6.22, p = .013).

**Figure S1.** Differences in Hours of Use: Arm x Time**.**

**
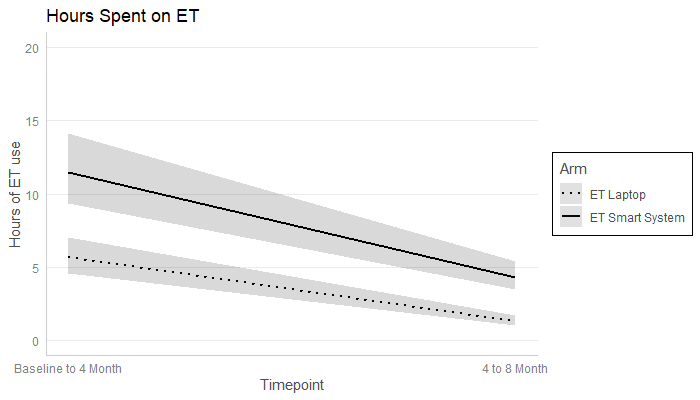
**

|  |  |  |  |  |  |  |  |  |  |
| --- | --- | --- | --- | --- | --- | --- | --- | --- | --- |

**Figure S2:** Days of use Arm x Time for Discussion Group.

**
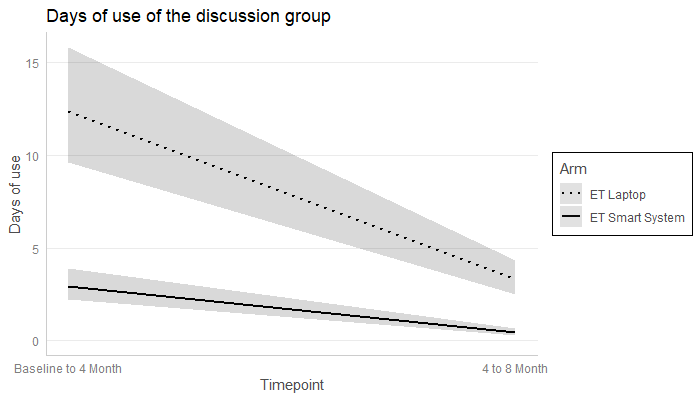
**

**Figure 7: Days of**

**Figure S3:** Days of use Arm x Time for Health Library.

**
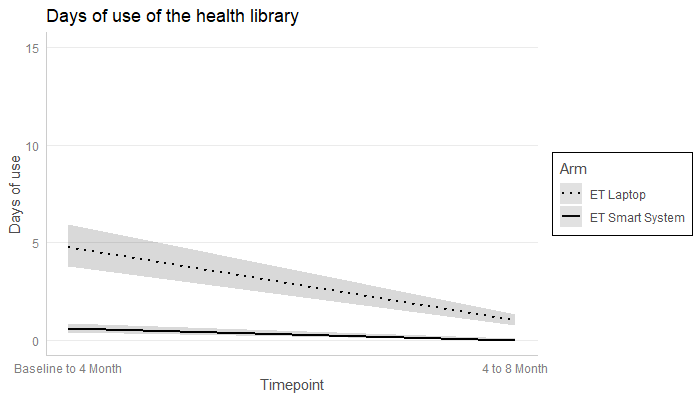
**

**Table S2.** Secondary Outcomes by Study Arm and Time.

| **outcome** | **Predictors** | **Estimates** | **CI** | **p** | **Estimate type** |
| --- | --- | --- | --- | --- | --- |
| Physical QOL  PROMIS | Time * Arm LT+SD vs C | 0.20 | -0.44 – 0.85 | .54 | Estimates |
|  | Time * Arm LT vs SD | -0.63 | -1.38 – 0.12 | .10 |  |
| Pain Intensity PROMIS | Time * Arm LT+SD vs C | 0.07 | -0.17 – 0.30 | .58 |  |
|  | Time * Arm LT vs SD | -0.16 | -0.43 – 0.12 | .26 |  |
| 30-day readmissions to hospital | Time * Arm LT+SD vs C | 0.30 | 0.06 – 1.59 | .16 | Odds Ratios |
|  | Time * Arm LT vs SD | 1.37 | 0.26 – 7.11 | .71 |  |
| Health Distress | Time * Arm LT+SD vs C | 0.05 | -0.09 – 0.18 | .50 | Estimates |
|  | Time * Arm LT vs SD | 0.13 | -0.03 – 0.28 | .10 |  |
| Well-being | Time * Arm LT+SD vs C | 0.04 | -0.53 – 0.61 | .89 |  |
|  | Time * Arm LT vs SD | -0.47 | -1.13 – 0.20 | .17 |  |
| Loneliness PROMIS | Time * Arm LT+SD vs C | -0.46 | -1.64 – 0.73 | .45 |  |
|  | Time * Arm LT vs SD | 1.03 | -0.35 – 2.41 | .14 |  |
| Irritability | Time * Arm LT+SD vs C | -0.06 | -0.49 – 0.37 | .78 |  |
|  | Time * Arm LT vs SD | 0.18 | -0.32 – 0.69 | .47 |  |
| Communication with Physicians | Time * Arm LT+SD vs C | 0.10 | -0.07 – 0.27 | .26 |  |
|  | Time * Arm LT vs SS | -0.12 | -0.32 – 0.07 | .22 |  |

**Table S3.** Exploratory Outcomes by Study Arm and Time.

| **Outcome** | **Predictors** | **Estimates** | **CI** | **p** | **Estimate type** |
| --- | --- | --- | --- | --- | --- |
| Alcohol  Use | Time * Arm LT+SD vs C | 0.98 | 0.85 – 1.14 | .83 | Incidence Rate Ratios |
|  | Time * Arm LT vs SD | 1.02 | 0.86 – 1.21 | .79 |  |
| Cigarette Use (yes/no) | Pearson’s Chi-squared test | 1.66 | NA | .44 | Chi-square |
| Falls (yes/no) | Time * Arm LT+SD vs C | 0.67 | 0.27 – 1.67 | .39 | Odds Ratios |
|  | Time * Arm LT vs SD | 0.79 | 0.28 – 2.21 | .66 |  |
